# Oncogenic Ras triggers hyperproliferation and impairs polarized colonic morphogenesis by autocrine ErbB3 signaling

**DOI:** 10.18632/oncotarget.10658

**Published:** 2016-07-18

**Authors:** Yvonne Möller, Markus Morkel, Jens Schmid, Sven Beyes, Janina Hendrick, Michaela Strotbek, Pamela Riemer, Simone Schmid, Lisa C. Schmitt, Roland Kontermann, Thomas Mürdter, Matthias Schwab, Christine Sers, Monilola A. Olayioye

**Affiliations:** ^1^ Institute of Cell Biology and Immunology, University of Stuttgart, Stuttgart, Germany; ^2^ Laboratory of Molecular Tumor Pathology and Systems Biology, Institute of Pathology, Charité Universitätsmedizin Berlin, Berlin, Germany; ^3^ Dr. Margarete Fischer-Bosch Institute of Clinical Pharmacology and University of Tübingen, Stuttgart, Germany; ^4^ DKTK, German Cancer Consortium, Partner site Charité, Berlin, Germany; ^5^ Stuttgart Research Center Systems Biology (SRCSB), University of Stuttgart, Stuttgart, Germany; ^6^ Department of Clinical Pharmacology, University Hospital, Tübingen, Germany; ^7^ Department of Biochemistry and Pharmacy, University of Tübingen, Tübingen, Germany; ^8^ Current address: Institute of Molecular Medicine and Cell Research, Faculty of Biology, Albert-Ludwigs-University, Freiburg, Germany

**Keywords:** oncogenic Ras, ErbB3/HER3, 3D culture, intestinal organoids, apical-basolateral polarity

## Abstract

Here we study the effects of inducible oncogenic K-Ras (G12V) expression on the polarized morphogenesis of colonic epithelial cells. We provide evidence that the autocrine production of heregulins, ligands for the ErbB3 receptor tyrosine kinase, is responsible for the hyperproliferation and aberrant 3D morphogenesis upon oncogenic K-Ras expression. This is in line with results obtained in primary intestinal organoid cultures, in which exogenous heregulin is shown to interfere with normal tissue architecture. Importantly, ErbB3 inhibition and heregulin gene silencing rescued K-Ras^G12V^-induced features of cell transformation. Together with the increased ErbB3 positivity detected in human high-grade primary colorectal cancers, our findings provide support for an autocrine signaling loop engaged by oncogenic K-Ras involving ErbB3 that contributes to the dedifferentiation of the intestinal epithelium during tumor initiation and progression.

## INTRODUCTION

Colorectal cancer (CRC) originates from the neoplastic transformation of epithelial cells, progressing from an adenoma to a carcinoma stage [1]. In approximately 40% of CRC cases, somatic missense mutations in codons 12, 13, or 61 of *K-Ras* impair its GTPase activity, resulting in constitutive activation of the protein [1–3]. In general, this is thought to uncouple Ras activation from extracellular signaling cues such as growth factor binding to receptor tyrosine kinases (RTKs), thereby conferring growth factor independence to mutant cells. Therefore, tumors with mutated *K-Ras* fail to respond to Cetuximab and Panitumumab, monoclonal antibodies (mAbs) targeting the epidermal growth factor receptor (EGFR/ErbB1). Anti-ErbB1 therapy is thus restricted to patients with no detectable *K-Ras* mutation and no targeted therapies are currently available for patients with *K-Ras* mutant CRC [4, 5].

ErbB1 belongs to the ErbB family of RTKs, which further comprises ErbB2/HER2, ErbB3/HER3 and ErbB4/HER4. Upon binding of specific peptide ligands the receptors homo- and heterodimerize, triggering tyrosine phosphorylation of the cytoplasmic tails and activation of downstream signaling. This includes activation of the Ras proteins, and subsequently the MAPK and PI3K pathways, which mediate biological responses such as proliferation, invasion and survival [6]. Although ErbB2 has no direct ligand, it readily dimerizes with the other ErbB receptors due to its constitutively active conformation [7]. ErbB3 is unique in that it has an impaired kinase domain, but in a heterodimer with a signaling competent ErbB family member, ErbB3 becomes phosphorylated and can serve as a signaling platform [8, 9]. The presence of several consensus sites for the p85 subunit of PI3K mediates the potent induction of PI3K-Akt signaling by phosphorylated ErbB3 [8, 10].

ErbB receptors are activated by a variety of different peptide ligands. Whereas EGF, TGF-α and amphiregulin bind to ErbB1, the heregulins (HRGs; also known as neuregulins) bind ErbB3 and ErbB4 [11, 12]. In epithelia that express both, the ligands and receptors, tight junctions separate the different subcellular membranes the receptors and cognate ligands are directed to, thereby preventing autocrine stimulation [13]. In cancer, different mechanisms can contribute to autocrine signals: firstly, cell polarity and therefore the separation between apical and basolateral membranes of epithelial cells can be compromised [14], and secondly, tumor-specific changes in gene expression can result in the complementation of cognate ligand-receptor pairs in the transformed tissue [15, 16].

Studies in cell culture have been instrumental in delineating tumor-associated signaling pathways and genetic alterations on cellular behavior, however, classical monolayer cultures do not replicate the complex interactions of the apical and basolateral membrane compartments. By contrast, cultivation of epithelial cells in a three-dimensional (3D) environment containing extracellular matrix (ECM) components recapitulates some of the conditions found *in vivo* [17, 18]. In such culture systems the establishment and maintenance of polarized morphology can be studied and the different steps of tumor initiation and progression modeled. More recently, the development of 3D intestinal organoid cultures derived from primary tissues has enabled the study of differentiation programs and epithelial tissue organization *ex vivo* [19].

Here we investigate how the acute expression of oncogenic K-Ras^G12V^ disrupts polarized morphogenesis of colonic epithelial cells in 3D culture and identify a novel autocrine signaling loop that mediates hyperproliferation and loss of cell polarity involving the RTK ErbB3. We moreover show that exogenous HRG addition is sufficient to mimic these effects both in Caco-2 CRC cells and in primary intestinal organoid cultures. Our findings thus have implications for the development of anti-cancer therapies targeting the HRG-ErbB3 signaling axis in the context of *K-Ras* mutant CRC.

## RESULTS

**Figure 1:**
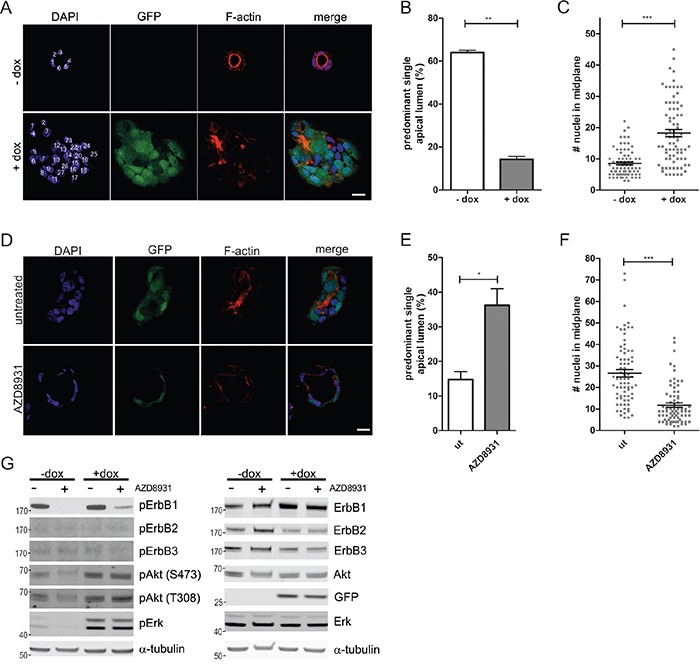
ErbB receptor inhibition restores aberrant morphogenesis of Caco-2 K-Ras^G12V^ cells in 3D culture **A.** Caco-2tet K-Ras^G12V^ cells were seeded into 3D cultures in the absence or presence of dox. Three days post seeding lumen expansion was induced by CTX. Cultures were fixed two days later and stained with DAPI (nuclei; blue) and phalloidin (F-actin; red). GFP is co-expressed with K-Ras^G12V^ (green). Shown are confocal sections of the midplane of representative cysts, nuclei are numbered in white (scale: 20 μm). **B.** The percentage of cysts with PSAL from (A) was determined (n>70; N=3). **C.** The number of nuclei in the midplane of cysts from (A) was counted (n=25; N=3). **D.** Caco-2tet K-Ras^G12V^ cells were seeded into 3D cultures. One day post seeding K-Ras^G12V^-expression was induced by dox and cultures were left untreated or treated with 200 nM AZD8931. Two days later CTX was added. Cultures were fixed the next day and stained with DAPI (nuclei; blue) and phalloidin (F-actin; red). GFP is co-expressed with K-Ras^G12V^ (green). Shown are confocal sections of the midplane of representative cysts (scale: 20 μm). **E.** The percentage of cysts with PSAL from (D) was determined (n>60; N=3). **F.** The number of nuclei in the midplane of cysts from (D) was counted (n=25; N=3) **G.** Caco-2tet K-Ras^G12V^ cells were seeded into 3D cultures. One day post seeding K-Ras^G12V^ expression was induced by dox and cultures were left untreated or treated with 200 nM AZD8931. Lysates were generated four days after seeding and analyzed by immunoblotting using the indicated antibodies (pErbB2(Tyr1248)). Tubulin was detected as a loading control. A representative blot is shown (N=3).

The human CRC cell line Caco-2 forms polarized cysts when grown in 3D matrigel cultures, recapitulating morphological features of the intestinal epithelium. These cysts are characterized by a single epithelial cell layer with apical-basolateral polarity that surrounds a hollow lumen [20]. Doxycycline-inducible expression of oncogenic K-Ras^G12V^ in these cells leads to the formation of hyperproliferating spherical structures, which are no longer polarized and fail to establish a central lumen (Figure 1A) [21, 22]. Quantification of the effects of K-Ras^G12V^ expression, visualized by the bi-cistronic GFP expression, showed that the average number of cells in the midplane of the cysts doubled compared to the non-induced control and only ~15% of the cysts were scored as normal, based on morphological criteria (see methods for details) that include well-defined apical F-actin accumulation (Figure 1B, 1C).

To investigate the potential contribution of the ErbB receptors to the aberrant morphogenesis and hyperproliferation induced by K-Ras^G12V^ expression, we treated the doxycycline-containing 3D cultures with the pan-ErbB inhibitor AZD8931. This inhibitor potently suppressed short-term ligand-induced ErbB receptor activation and downstream signaling in Caco-2 cells (Supplementary Figure S1A). Interestingly, the abnormal morphology and hyperproliferation of K-Ras^G12V^-expressing Caco-2 cysts was partially restored, with almost 40% of cysts displaying polarized structures with a predominant single apical lumen (PSAL; Figure 1D-1F). Next, we recovered the cysts from the 3D cultures, and analyzed the lysates by immunoblotting. Compared to the low basal phosphorylation in non-induced cells (-dox), K-Ras^G12V^ expression (+dox) revealed hyperactivation of MAPK and PI3K pathways, as judged by increased phosphorylation of Erk and Akt on T202/Y204 and T308/S473, respectively (Figure 1G). ErbB1 phosphorylation was readily detectable in both control and K-Ras^G12V^-expressing cells, whereas no phosphorylation was visible for ErbB2 and ErbB3. Expression of ErbB4 was below the detection limit. AZD8931 potently suppressed ErbB1 phosphorylation in control (-dox) and oncogenic Ras-expressing cells. Although AZD8931 addition to the cultures three days before harvest suppressed Erk and Akt phosphorylation in the control cells, Erk and Akt phosphorylation were maintained in K-Ras^G12V^-expressing cells, illustrating the dominant signaling of oncogenic K-Ras with respect to the MAPK and PI3K pathways (Figure 1G). Comparable results were obtained with a second inhibitor (ErbB2-II), which also restored the morphogenetic defects in Caco-2tet Ras^G12V^ cells (Supplementary Figure S1B, left). This inhibitor blocked HRG-induced ErbB2 and ErbB3 activation, but had no effect on EGF-induced ErbB1 phosphorylation (Supplementary Figure S1B, right).

**Figure 2:**
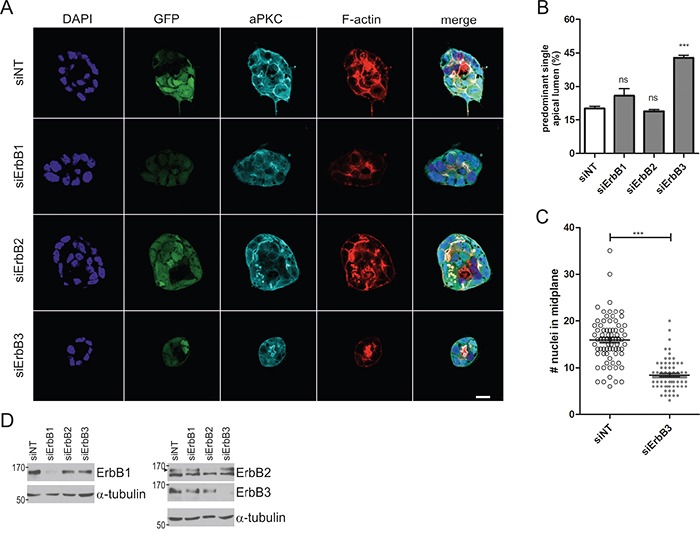
ErbB3 knockdown rescues hyperproliferation and polarized morphogenesis of Caco-2 cells expressing oncogenic K-Ras **A.** Caco-2tet K-Ras^G12V^ cells were transfected with non-targeting (siNT) and ErbB receptor-specific siRNAs, respectively. The next day cells were seeded into 3D culture. One day post seeding K-Ras^G12V^ expression was induced with dox. Two days later CTX was added. Cultures were fixed the next day and stained with DAPI (nuclei; blue), anti-aPKC antibody (cyan) and phalloidin (F-actin; red). GFP is co-expressed with K-Ras^G12V^ (green). Shown are confocal sections of the midplane of representative cysts (scale: 20 μm). **B.** The percentage of cysts with PSAL from (A) was determined (n>70; N=3). **C.** The number of nuclei in the midplane of cysts from (A) was counted (n=25; N=3). **D.** Two days after gene silencing, lysates were generated and analyzed by immunoblotting using the indicated antibodies. Tubulin was detected as a loading control. Specific bands are marked by arrowheads.

To identify the ErbB receptors that cooperate with K-Ras^G12V^ signaling, we performed gene silencing experiments. Caco-2tet K-Ras^G12V^ cells were transfected with control siRNA (siNT) or ErbB receptor-specific siRNAs (siErbB1/2/3) and then seeded into 3D culture, followed by the induction of K-Ras^G12V^ the next day. Morphological analysis revealed that the cysts formed by oncogenic Ras-expressing cells depleted of ErbB1 or ErbB2 were comparable to those transfected with the siRNA control. By contrast, upon ErbB3 depletion, the K-Ras^G12V^-induced defect in polarized morphogenesis was rescued, as seen by the apical accumulation of the marker atypical PKC (aPKC) and F-actin (Figure 2A, 2B). Note that K-Ras^G12V^ expression was not altered by the siRNA transfection as judged by the GFP signal. Additionally, in the absence of ErbB3, the hyperproliferation triggered by K-Ras^G12V^ was abrogated (Figure 2C), with average cell numbers in the midplane of cysts comparable to those observed in non-induced control cultures (Figure 1C). This was specific to the 3D cultures, as ErbB3 depletion did not inhibit the proliferation of Ras^G12V^-expressing cells grown in 2D monolayers (data not shown). ErbB receptor knockdown was verified by immunoblotting (Figure 2D) and similar results were obtained by independent siRNAs (Supplementary Figure S2A+S2B). Analysis of downstream signaling showed no impact of ErbB3 depletion on the Ras^G12V^-dependent activation of Erk and Akt (Supplementary Figure S2C). Taken together, these findings uncover a role for ErbB3 in oncogenic Ras-induced hyperproliferation and polarity loss, which were partially rescued by pharmacologic pan-ErbB receptor inhibition or specific siRNA-mediated knockdown.

**Figure 3:**
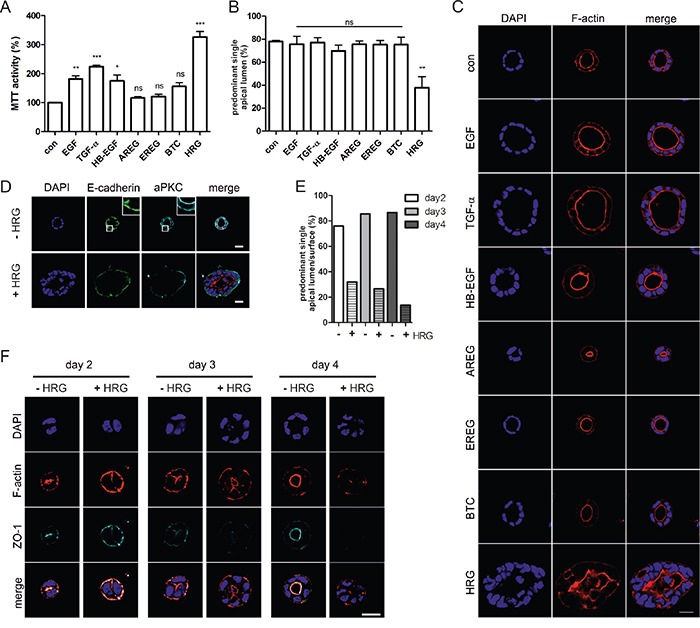
Heregulin impairs apical-basolateral polarity and lumenogenesis of Caco-2 cysts **A.** Caco-2 cells were grown in 3D cultures containing 2% FCS in the presence of the indicated growth factors (10 ng/ml) or in 2% FCS as a control (con). 3D cultures were analyzed by MTT assay at day 6 (N=3).(B+C) Cells were grown as described in (A). Three days later CTX was added. Cultures were fixed the next day and stained with DAPI (nuclei; blue) and phalloidin (F-actin; red). **B.** The percentage of cysts with PSAL was determined (n>70; N=3). **C.** Shown are confocal sections of the midplane of representative cysts (scale: 20 μm). **D.** Caco-2 cells grown in the presence or absence of HRG were stained with anti-E-cadherin antibody (green), anti-aPKC antibody (cyan) and DAPI (nuclei; blue). (E+F) Caco-2 cells were grown in 3D cultures containing 10% FCS in the presence or absence of HRG (10 ng/ml). Cultures were fixed at the indicated days after seeding, and stained with DAPI (nuclei; blue) and phalloidin (F-actin; red). At day three, CTX was added. **E.** The percentage of cysts with PSAL was determined (n>25; N=2). **F.** Shown are confocal sections of the midplane of representative cysts (scale: 20 μm).

To analyze the impact of different ErbB ligands on Caco-2 3D proliferation and morphogenesis, we embedded the parental cells in growth factor-reduced matrigel with low serum (2%) in the presence of the different ErbB ligands. Stimulating effects as measured by MTT activity were detected following EGF, TGF-α, and HB-EGF addition and strongest stimulation was induced by HRG (Figure 3A). Importantly, despite effects on proliferation as seen by the increased sizes of cysts (Figure 3C), apart from HRG, none of the ErbB ligands interfered with polarized cyst morphology and lumenogenesis (Figure 3B, 3C). In the presence of HRG, a significant drop in the percentage of cysts with defined apical F-actin staining and central lumens was noted (Figure 3B, 3C), similar to the phenotype induced by K-Ras^G12V^. Expression of aPKC and E-cadherin, which demarcate the apical and basolateral membranes, respectively, were retained in the presence of HRG, but distinct apical aPKC localization was lost (Figure 3D). To prove that HRG signaling was disrupting apical-basolateral polarity and not simply inducing hyperproliferation leading to luminal filling, we analyzed cyst development in more detail (Figure 3E, 3F). Already at the two-cell stage, apical-basolateral polarization is observed, with F-actin accumulating at the cell-cell interface, associated with the formation of tight junctions that are positive for ZO-1 (Figure 3F; day 2, -HRG). This apical surface is maintained as the cyst develops, specifying the position of the lumen. HRG interfered with correct F-actin localization and ZO-1 recruitment, indicating that the initial establishment of polarity and tight junctions were impaired (Figure 3E, 3F).

**Figure 4:**
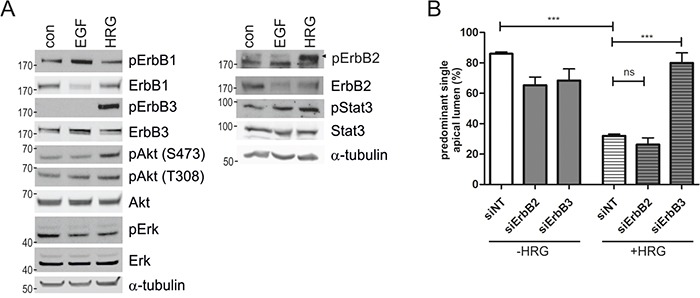
HRG-induced ErbB3 signaling impairs polarized morphogenesis of Caco-2 cells **A.** Caco-2 cells were grown in 3D cultures containing 2% FCS in the presence of the indicated growth factors (10 ng/ml) or in 2% FCS as a control (con). Three days post seeding cysts were lysed and analyzed by immunoblotting using the indicated antibodies (pErbB2(Tyr1248)). Tubulin was detected as a loading control. Shown is a representative blot (N=2). **B.** Caco-2 cells were transfected with non-targeting (siNT) and ErbB-receptor specific siRNAs, respectively. The next day, cells were seeded into 3D culture in the presence or absence of HRG (10 ng/ml). Three days post seeding lumen expansion was induced by CTX. The next day, cultures were fixed and stained with DAPI and phalloidin (not shown). The percentage of cysts with PSAL was determined (n>50; N=3).

Biochemical analysis of cell lysates derived from Caco-2 3D cultures grown in the presence of EGF or HRG for three days revealed that EGF induced ErbB1 phosphorylation, whereas HRG induced the phosphorylation of ErbB2 and ErbB3 (Figure 4A). Erk phosphorylation was not maintained after long-term stimulation, whereas Stat3 phosphorylation was slightly elevated in the presence of the ligands. Compared to EGF, HRG led to sustained Akt phosphorylation (3-fold) at serine 473 (Figure 4A), which was comparable to the extent of Akt phosphorylation induced by K-Ras^G12V^ expression in the Caco-2tet cells (Figure 1C). Finally, to prove that ErbB3 was mediating the HRG effect, we knocked down ErbB3 and also ErbB2, as ErbB3 preferentially heterodimerizes with ErbB2, followed by seeding of cells into 3D cultures. In the absence of HRG, the receptor knockdowns had little effect on the polarized morphogenesis of the control cultures (siNT) (Figure 4C, left). However, in the presence of HRG, ErbB3 depletion fully restored polarized cyst structure and lumen formation to the level seen in the control. Knockdown of ErbB2, however, failed to do so (Figure 4C, right).

**Figure 5:**
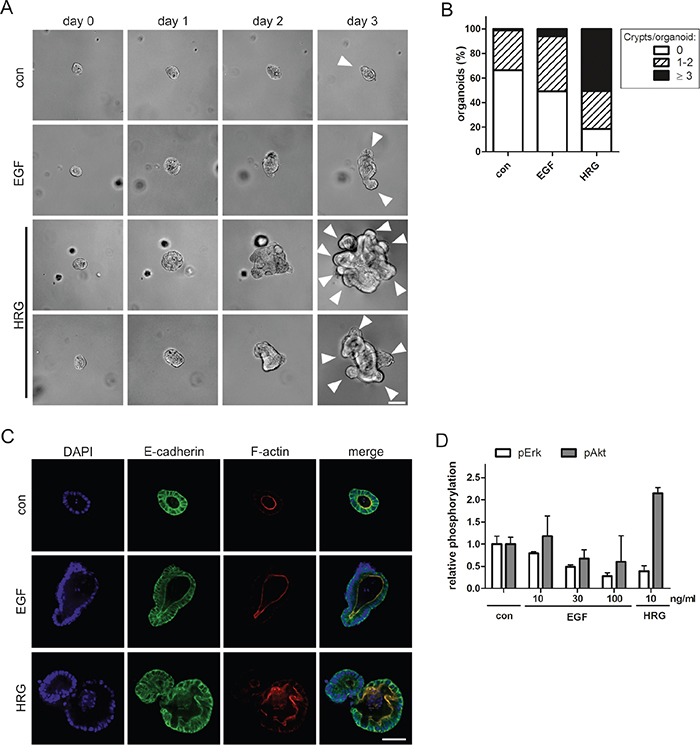
HRG triggers hyperproliferation and disrupts the architecture of intestinal organoids **A.** Representative photographic series of intestinal crypts developing in the absence (con) or presence of 10 ng/ml EGF or HRG in 3D organoid culture. White arrowheads on day 3 indicate crypt domains. (Scale: 100 μm). **B.** Quantification of crypt domains per organoid on day three (N=3). **C.** Organoids were fixed on day 1 and stained with anti-E-cadherin antibody (green), phalloidin (F-actin; red) and DAPI (nuclei; blue) (scale: 50 μm). **D.** Capillary phospho-protein analysis of untreated organoids (con) and treated with EGF or HRG (N=2).

To determine whether the distinct morphological phenotypes induced by EGF and HRG could be recapitulated in normal intestinal epithelium, we analyzed these ligands in 3D murine intestinal organoid cultures. In accordance with ErbB receptor expression in Caco-2 cells, intestinal epithelium from wildtype and APC^min^ mice expressed *ErbB1, ErbB2* and *ErbB3*, but lacked detectable transcript levels of *ErbB4* (Supplementary Figure S2A). We found that the outgrowth of single intestinal crypts into organoids with a crypt-villus axis was stalled in the absence of ErbB1 ligands. EGF supported the outgrowth of organoids with 1-2 crypt domains within 3-4 days, as reported previously [19]. However, when we exchanged EGF for HRG, the crypts formed hyperproliferative organoids with multiple irregularly shaped crypts within 2-3 days (Figure 5A, 5B). Immunofluorescence stainings revealed morphogenetic defects in the presence of HRG, visible as epithelial tufting with partial loss of apical localization of F-actin (Figure 5C). Phospho-protein analysis of the organoids revealed sustained Akt activation by HRG, but not by EGF (Figure 5D). Even high doses of EGF did not upregulate Akt, indicating that the differential signaling induced by EGF and HRG is qualitative and not quantitative. Reminiscent of the Caco-2 model (Figure 4B), long-term stimulation with EGF or HRG suppressed Erk activation (Figure 5D), probably owing to compensatory feedback mechanisms. This analysis of normal tissue provides support that activation of HRG-ErbB3 signaling can contribute to hyperproliferation and tissue disorganization in the intestinal epithelium.

**Figure 6:**
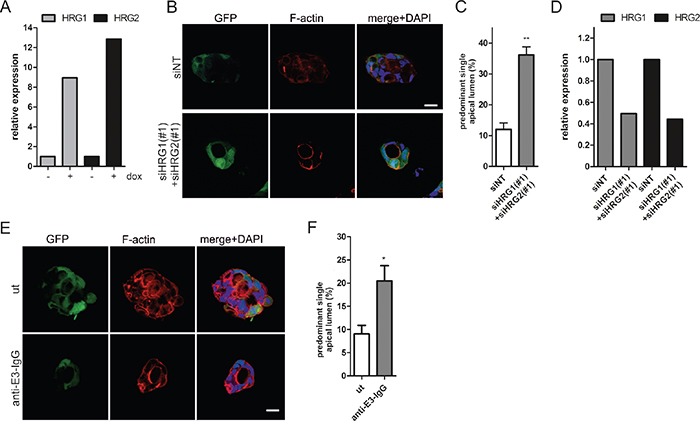
HRG depletion restores lumen formation in oncogenic K-Ras^G12V^ expressing Caco-2 cells **A.** Caco-2tet K-Ras^G12V^ cells were seeded into 3D in the presence of dox. Three days after seeding, cysts were isolated, RNA was extracted, and *HRG1/HRG2* expression was determined by qPCR and normalized to *GAPDH* (N=2). (B-D) Caco-2tet K-Ras^G12V^ cells were transfected with non-targeting (siNT) and a mix of HRG1- and HRG2-specific siRNAs (#1). The next day cells were seeded into 3D culture and K-Ras^G12V^ expression was induced with dox one day later. **B.** Cultures were fixed three days later and stained with DAPI (nuclei; blue), anti-aPKC antibody (cyan) and phalloidin (F-actin; red). GFP is coexpressed with K-Ras^G12V^ (green). Shown are confocal sections of the midplane of representative cysts (scale: 20 μm). **C.** The percentage of cysts with PSAL from (B) was determined (n>70; N=3). **D.** Cysts were isolated three days later and RNA was extracted. *HRG1/HRG2* expression was determined by qPCR and normalized to *GAPDH* (N=2). **E.** Cells were seeded into dox-containing 3D culture in the absence (ut) or presence of 100 nM anti-E3-IgG. Three days later CTX was added. Cultures were fixed the next day and stained with DAPI (nuclei; blue), anti-aPKC antibody (cyan) and phalloidin (F-actin; red). GFP is coexpressed with K-Ras^G12V^ (green). Shown are confocal sections of the midplane of representative cysts (scale: 20 μm). **F.** The percentage of cysts with PSAL from (E) was determined (n>70; N=3).

Based on our findings, we hypothesized that K-Ras^G12V^ expression in Caco-2 3D cultures may lead to enhanced HRG production. Indeed, real-time qPCR analysis revealed that oncogene expression induced *HRG1/2* transcript levels (Figure 6A, 6B). We next sought to silence HRG expression prior to the induction of oncogenic Ras. In full support of our hypothesis, the combined depletion of HRG1 and HRG2 reduced spheroid size and significantly increased the number of polarized cysts with apical lumens (Figure 6B, 6C). This rescue was also observed by independent siRNAs (Supplementary Figure S3) and was comparable to the rescue obtained by pharmacological ErbB inhibition (Figure 1E) or ErbB3 silencing (Figure 2B). Efficient *HRG1/2* knockdown in Ras^G12V^-expressing cells isolated from the 3D cultures is shown in Figure 6D. To obtain proof that HRG was acting through extracellular ErbB3 binding, we treated Ras^G12V^-expressing cells with an ErbB3-specific antibody that competes with HRG. Importantly, this treatment suppressed proliferation (data not shown) and significantly restored the number of cysts with a normal polarized morphology (Figure 6E, 6F). In conclusion, the hyperproliferation and polarity loss induced by K-Ras^G12V^ expression in the 3D Caco-2 model can be ascribed to autocrine HRG-ErbB3 signaling.

Compared to human CRC cell lines with wildtype *K-Ras* genes, the analysis of *HRG* expression in cell lines harboring activating *K-Ras* mutations revealed strongly increased *HRG2* transcript levels in three out of four cases (SW480, LoVo, HCT116; Supplementary Figure S4C). This indicates that the upregulation of HRG expression by oncogenic Ras signaling is not restricted to the Caco-2 model system. We further found that several ErbB ligands including *HRG1* were upregulated in mouse adenoma compared to normal intestinal tissue (Supplementary Figure S4B), pointing at the existence of further possible mechanisms activating ErbB receptor signaling during tumor initiation and progression.

**Figure 7:**
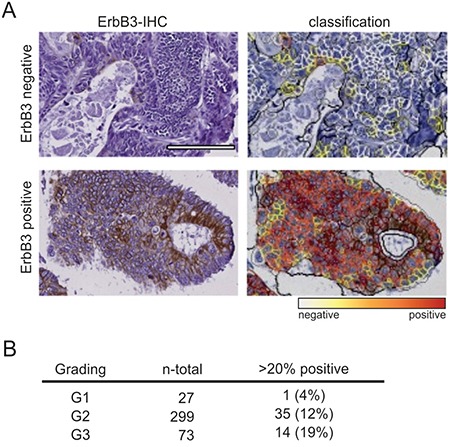
ErbB3 expression correlates with tumor grade in primary colon cancers **A.** ErbB3 staining of two representative specimens (left) and computer-based quantification of staining intensity by Definiens software (right). Top: ErbB3 negative; bottom: ErbB3 positive. (Scale: 100 μm). **B.** Total numbers and percentages of G1, G2 and G3 specimens with more than 20% ErbB3-positive cells are listed.

Finally, by IHC staining of TMAs, we analyzed the expression of ErbB3 in 399 colon tumor samples. Staining intensity was quantified in an automated manner and cells with medium or high expression were scored as ErbB3 positive (Figure 7A). Comparison of specimens with less than 20% ErbB3-positive cancer cells with those harboring more than 20% ErbB3-positive cells revealed a significant difference in tumor grade, as determined by chi square trend test (p=0.025). Whereas only a single G1 tumor contained more that 20% ErbB3-positive cells (1/27; 4%), 12% of the G2 tumors (35/299) and 19% of the G3 tumors (14/73) displayed medium-to-strong ErbB3 expression in more than 20% of the cells (Figure 7B). This correlation between ErbB3 positivity and increasing tumor grade provides additional support for the contribution of ErbB3 signaling to the dedifferentiation of the intestinal epithelium during tumor progression.

## DISCUSSION

Using a 3D CRC cell culture model, we here show for the first time that K-Ras^G12V^ induced hyperproliferation and loss of polarized morphogenesis, both of which are hallmarks of tumor progression, engage a HRG/ErbB3 dependent signaling loop. In CRC, *Ras* mutations are preceded by mutations in *APC* associated with the activation of the WNT pathway and the formation of benign polyps [23]. Considering the frequent co-occurrence of *APC* and *K-Ras* mutations, Caco-2 cells are a suitable model for the analysis of the early molecular and cellular changes induced by Ras activation, as these cells harbor an inactivating mutation in *APC*, but they lack oncogenic alterations of the *Ras* or *Raf* genes [21].

Despite its impaired kinase activity, ErbB3 supports cell survival and mediates drug resistance [8, 10], primarily through the potent induction of the PI3K pathway. In breast cancer, the ErbB2-ErbB3 signaling dimer was shown to be essential for tumor formation and maintenance [24–26]. Recently, somatic mutations in ErbB3 with transforming potential were reported in colon and gastric cancers, however, these variants were still functionally dependent on ErbB2 [27]. Although ErbB2-ErbB3 is regarded as the most potent ErbB dimer, ErbB3 also dimerizes with ErbB1 and non-ErbB family RTKs such as c-Met [28]. An intestinal-specific *ErbB3* knockout mouse model showed that the loss of ErbB3 resulted in loss of the ErbB4 protein, indicative of the formation of active ErbB3/4 heterodimers [29]. Interestingly, when crossed to Apc^Min^ mice, ErbB3 loss almost completely prevented colonic tumorigenesis, underscoring the important role of ErbB3 signaling in tumor establishment and progression [29]. The promiscuous pairing of ErbB3 with other RTKs may explain why in our experiments the single knockdown of ErbB1 or ErbB2 did not mirror the effects of ErbB3 depletion. We also cannot rule out that residual protein in siRNA-treated cells is sufficient for ErbB3 transactivation, in line with disproportionate ErbB2 levels found to efficiently transactivate ErbB3 [30]. The fact that ErbB3 phosphorylation was below the detection limit upon K-Ras^G12V^ induction in Caco-2 cells further indicates that a low number of activated receptors suffices in the cooperative signaling with oncogenic Ras.

Mutant K-Ras has been associated with the increased expression and/or shedding of ErbB1 ligands [15, 16, 31]. Here we show that K-Ras^G12V^ expression induces the transcriptional activation of *HRG1/2*, the ablation of which normalized the proliferation and morphology of Caco-2 cells. By exogenous addition of HRG to Caco-2 and primary intestinal organoid cultures we confirmed that HRG-induced ErbB3 signaling is not compatible with intestinal epithelial organization. Addition of ErbB1 ligands enhanced the proliferation of Caco-2 and organoid cultures but did not interfere with polarized morphogenesis. This highlights (i) the independent regulation of proliferation and polarity and (ii) the qualitatively distinct signaling characteristics of the different ErbB ligands.

Considering that Akt activation is strongly suppressed in Caco-2 3D cultures compared to 2D monolayers [22], the elevated pAkt levels downstream of ErbB3 may contribute to the HRG-induced proliferation and/or polarity loss. However, in a previous report, the stable expression of activated PI3K in Caco-2 cells did not perturb 3D morphogenesis and PI3K inhibition failed to rescue the morphology or size of Caco-2 spheroids stably expressing mutant Ras [21]. This suggests that further signaling pathways besides PI3K are required to stimulate proliferation and disrupt epithelial morphogenesis in Caco-2 Ras^G12V^ cells. For example, in pancreatic adenocarcinoma, both PI3K and Stat3 activation downstream of ErbB1 were found to be essential for Ras-induced transformation [32]. In Madin-Darby canine kidney cells the specific inhibition of the aPKC isoform PKCiota partially restored the non-polar spheroid morphogenesis observed in cells overexpressing activated H-Ras or ErbB2 [33]. In epithelial cells, spatially controlled aPKC activity is necessary for the establishment and maintenance of apicobasal polarity. Considering that the recruitment of the Par6-aPKC complex to activated ErbB2 in the MCF10A breast epithelial acini model was shown to mediate disruption of epithelial polarity [34], it is tempting to speculate that HRG-activated ErbB3 in intestinal cells may operate through a similar mechanism.

Increased expression of ErbB3 in CRC was found to correlate with poorer overall survival and disease stage [35, 36] and as revealed in this study with higher tumor grade. However, there is still little known about the expression of ErbB3 and the ErbB ligands in the different CRC subtypes. Irrespective of cancer-associated mutations in *K-Ras*, aberrant HRG production by the stromal compartment could also impair the integrity of the intestinal epithelium. Apart from driving proliferation, HRG is a stimulator of cell migration and invasion [11, 12]. In future studies it will be important to analyze the signaling profiles and transcriptional programs activated in the epithelial cells responding to this ligand.

Currently, several ErbB3-directed antibodies targeting the extracellular domain of the receptor are in clinical development [37]. A subset of CRC patients with Ras wildtype tumors fails to show an initial response to anti-ErbB1 therapy, whereas those that do respond routinely develop resistance, often by selection of Ras mutant clones. In such patients elevated circulating HRG levels were associated with both *de novo* and acquired resistance to Cetuximab therapy [38]. Several reports describe the upregulation of ErbB3 as an evasion strategy to RTK inhibition [39–41] and pharmacologic MEK inhibition downstream of mutant Ras also unleashes feedbacks at the level of RTKs, including ErbB3 [42, 43]. Thus, combination treatments involving ErbB3 may be particularly effective for the treatment of Ras mutant CRC and may also prevent HRG-mediated resistance in Ras wildtype CRC.

## MATERIALS AND METHODS

### Antibodies and reagents

Antibodies used were monoclonal rabbit anti-pEGFR (Y1068), polyclonal rabbit anti-pERK (T202/Y204), monoclonal rabbit pErbB2 (Tyr1221/1222), polyclonal rabbit anti-pErbB2 (Tyr1248), monoclonal rabbit pErbB3 (Tyr1289), monoclonal mouse anti-ERK, monoclonal rabbit anti-pAKT (Thr308), monoclonal mouse anti-AKT (pan), monoclonal mouse anti-pAKT (Ser473), monoclonal mouse anti-Stat3 (124H6), monoclonal rabbit anti-pStat3 (Tyr705) (D3A7) XP^®^ (all from Cell Signaling, Danvers, MA, USA), polyclonal rabbit ZO-1 antibody (Invitrogen, Carlsbad, CA, USA), monoclonal mouse anti E-cadherin (BD, CA, SanJose, USA), monoclonal mouse anti-ErbB1 (Thermo Scientific, Fremont, MA, USA), polyclonal rabbit anti-aPKC (zeta), polyclonal rabbit anti-ErbB2, polyclonal rabbit anti-ErbB3 (all from Santa Cruz Biotechnology, Dallas, TX, USA) and monoclonal mouse anti-alpha-tubulin (Sigma-Aldrich, St Louis, MO, USA). The ErbB3-blocking antibody anti-E3-IgG was generated in-house and will be described in detail elsewhere (Schmitt et al., in preparation). HRP-labeled secondary anti-mouse and anti-rabbit IgG antibodies were from GE Healthcare (Buckinghamshire, UK). Alexa Fluor 488- and 546-labeled secondary anti-mouse and anti-rabbit IgG antibodies and Alexa Fluor 633-labeled phalloidin were from Invitrogen. DAPI was from Sigma-Aldrich. EGF was from R&D Systems Inc. (Minneapolis, MN, USA); TGF-α, HB-EGF, HRG were from PeproTech (Hamburg, Germany), and AREG, EREG, BTC were from ImmunoTools (Friesoythe, Germany). The pan-ErbB inhibitor AZD8931 (Sapitinib) was Absource Diagnostics (Munich, Germany). The ErbB2 Inhibitor II (#324732) was from Calbiochem (Darmstadt, Germany).

### Cell lines and 3D cell culture

Caco-2 cells were obtained from Interlab Cell Line Collection (Genova, Italy) in 2012 and reauthenticated by SNP analysis in 2016 (Multiplexion, Immenstadt, Germany). Caco-2tet cells, stably expressing the doxycycline-inducible system components rtTA and rtTS [44] and Caco-2tet K-Ras^G12V^ cells were described previously [22]. The corresponding parental cells were authenticated in 2014 (see above). SW480, LS174T, Colo-320DM and Difi cells were obtained from the Institute of Clinical Pharmacology (Stuttgart, Germany), Lovo and HCT116 cells were from Interlab Cell Line Collection (Genova, Italy). Caco-2, SW480, LS174T, Colo-320DM, Difi, Lovo and HCT116 cells were cultured in RPMI 1640 (Invitrogen) and Caco-2tet cells in DMEM (Invitrogen), both supplemented with 10% FCS (PAA Laboratories, Cölbe, Germany). Cell lines were incubated in a humidified atmosphere of 5% CO_2_ at 37°C. For growth in 3D, cells were seeded on a bed of growth factor reduced matrigel (BD) and PureCol^®^-S collagen (Advanced Biomatrix, San Diego, CA, USA) (1:1) and overlaid with growth medium containing 2% matrigel. Lumen expansion was induced by addition of 100 ng/ml Choleratoxin (CTX; Sigma Aldrich) at day 3 post seeding. Transgene expression in the Caco-2tet cells was induced by 2 μg/ml doxycycline (dox; Merck, Darmstadt, Germany). For growth factor dependent assays cells were grown in medium containing 2% FCS plus 10 ng/ml growth factor.

### MTT assays

Cells were seeded into matrigel/collagen-coated 96-well plates (100 μl medium containing 2% matrigel). Viability was determined by addition of 10 μl 3-(4,5-dimethylthiazol-2yl-)2,5-diphenyl tetrazolium (MTT; Roth, Karlsruhe, Germany) solution (5 mg/ml) followed by incubation for 3 h at 37°C. Cells were then lysed by addition of 100 μl 50% dimethylformamide containing 10% SDS and absorbance was measured at 570 nm using the multimode reader Infinite^®^ 200 PRO (Tecan, Männedorf, Switzerland).

### siRNAs

As non-targeting negative controls (siNT) ON-TARGETplus^®^ non-targeting control pool (Dharmacon) or Silencer^®^Select Negative Control#2 (Invitrogen) were used. In all cases, two independent siRNAs were used: siErbB1 refers to siGenome SMARTpool EGFR (M-003114-03-0005) from Dharmacon; siErbBR1#2 refers to Silencer^®^Select ErbB1 (s565) from Invitrogen); siErbB2 refers to a single duplex with the sense sequence: 5′-GGACGAAUUCUGCACAAUG-3′, from Eurofins MWG Operon; siErbB2#2 refers to Silencer^®^Select ErbB2 (s612) from Invitrogen; siErbB3 refers to siGenome SMARTpool ErbB3 (M-003127-03-0005) from Dharmacon; siErbBR3#2 refers to Silencer^®^Select ErbB3 (s4779) from Invitrogen); siHRG1#1 refers to Silencer^®^Select NRG1 (s230508), siHRG1#2 refers to Silencer^®^Select NRG1 (s230507), siHRG2#1 refers to Silencer^®^Select NRG2 (s18322) and siHRG2#2 refers to Silencer^®^Select NRG2 (s18321) (all from Invitrogen). Cells were transfected with siRNA using DharmaFECT1 (Dharmacon, Lafayette, CO, USA) according to manufacturer's instructions.

### Western blotting

Cells were lysed in RIPA buffer (50 mM Tris (pH 7.5), 150 mM NaCl, 1% Triton-X 100, 0.5 sodium deoxycholate, 0.1% SDS, 1 mM sodium orthovanadate, 10 mM sodium fluoride and 20 mM β-glycerophosphate plus Complete protease inhibitors (Roche, Basel, Switzerland). For lysates from 3D cultures, cells were cultured on pure matrigel without collagen. Spheroids were isolated after 4 days by dissolving the matrigel in ice cold Cell Recovery Solution (BD). After centrifugation the spheroids were lysed in RIPA buffer. Lysates were clarified by centrifugation. Equal amounts of protein were separated by SDS–PAGE (NuPAGE^®^ Novex Bis-Tris Gel; Invitrogen) and transferred to nitrocellulose membrane (iBlot^®^Gel Transfer Stacks; Invitrogen). Alternatively lysates were loaded on 10% polyacrylamide gels and transferred to polyvinylidene difluoride membranes (Roth). Membranes were blocked with 0.5% blocking reagent (Roche) in PBS containing 0.1% Tween-20 and incubated with primary antibodies, followed by HRP-conjugated secondary antibodies. Visualization was done with ECL detection system (Pierce, Rockford, IL, USA). Alternatively detection was done with IR-labled secondary antibodies IRDye 800 CW goat anti-mouse IgG (Licor Biotechnology, Bad-Homburg, Germany) and IRDye 680 LT goat anti-rabbit IgG (Licor Biotechnology).

### Quantitative PCR

Total RNA was isolated from cells using the RNeasy^®^ Plus Mini Kit (Qiagen, Foster City, CA, USA) according to the manufacturer's protocol. For 2D cell cultures, 2x10^5^ to 1x10^6^ cells were lysed with RLT buffer according to the manufacturer's protocol. For 3D culture, 4x10^5^ cells were seeded in 12-well plates on matrigel, as described for protein extraction. Pelleted spheroids were lysed in 350 μl RLT buffer according to the manufacturer's protocol. RNA samples were quantified using a Nanophotometer (Implen, Munich, Germany) at OD 260/280 nm. Q-PCR was performed with QuantiTect Primer Assays^®^ for SYBR^®^ Green-based expression analysis (Qiagen) using a Cfx96 device (Biorad) according to the manufacture's protocol for one-step RT-PCR. Primers used were Hs_NRG1_1_SG QuantiTect Primer Assay, Hs_NRG2_1_SG QuantiTect Primer Assay and Hs_GAPDH_2_SG QuantiTect Primer Assay (all Qiagen). Changes in the relative expression level were calculated using the 2-ΔΔCt method (Biorad CFX manager software 3.1.). *GAPDH* was used as the endogenous control gene for normalization.

### Immunofluorescence microscopy

Cells grown in 3D on matrigel/collagen coated 8-well glass chamber slides (BD) were fixed and stained as described [22]. To determine the number of cysts with a ‘predominant single apical lumen’ (PSAL) [33], spheroids were analyzed in terms of roundness, cell-free lumen formation and F-actin staining of the apical surface. Round spheroids with a single-cell layer around a hollow lumen and distinct F-actin staining were scored as normal, cysts lacking at least two of these features were scored as abnormal. For organoid stainings, organoids were washed with PBS to remove matrigel and fixed with 4% PFA, followed by immunostaining as described in [22]. Organoids were imaged in PBS on a cell observer spinning disc system (Zeiss) equipped with an oil immersion objective LD LCI Plan-Apochromat 25x/0.8 Imm Corr DIC M27 and an Axiocam 503 mono CCD camera using 405, 561 and 638 nm excitation. Images were processed with the ZEN software (Zeiss).

### Intestinal organoid cultures and protein analysis

Organoids of the murine small intestine were cultured as described [45]. For outgrowth analysis, organoids were washed in PBS, disaggregated into single crypts and embedded in growth factor-reduced matrigel and crypt culture medium containing Noggin (Peprotech, Rocky Hill, NJ, USA) and R-spondin (produced as described in [46]). Outgrowth in the absence or presence of EGF/HRG was monitored in 24 h intervals. Proteins were analyzed by a WES capillary Western system (ProteinSimple), using the 12-230 kDa Master Kit α-Rabbit-HRP and pAkt T308 and pErk1/2 T202/Y204 antibodies (1:50, Cell Signalling) and α-vinculin (1:30, Cell Signalling) for normalization. In short, organoids were washed in ice-cold PBS by centrifugation, lysed in 25 μl M-PER buffer (Pierce #78501) supplemented with PhosSTOP and Complete EDTA-free phosphatase inhibitor cocktails (Roche) for 30 min on ice, frozen at -80°C, thawed on ice, and sonified (Bioruptor/diagenode, 5 minutes at “H”, interval 0,5). Lysates were spun for 10 min at 4°C in a tabletop centrifuge, and protein was measured by BCA assay (Pierce). Lysates were used at 0.5 and 0.2 μg/μl for pAkt and pErk, respectively, according to the manufacturer's protocol.

### Tissue microarrays (TMAs) and immunohistochemical (IHC) analysis

Formalin-fixed paraffin-embedded (FFPE) colon cancer samples of 399 patients treated at the Robert Bosch Hospital were used for IHC analysis. Patient and clinicopathological data of colon tumors are given in Supplementary Table S1. The study was approved by the ethics committee in Tübingen. TMAs were prepared using the Manual Tissue Microarrayer MTA-1 (Beecher Instruments Inc, Sun Prairie, Wisconsin, USA) by punching three tissue cylinders (diameter: 600 μm) from each FFPE sample. For IHC analysis, TMAs were cut into 3 μm sections, incubated in EDTA buffer, pH 8.0 (30 min, steam heater). Staining for ErbB3 was done using anti-ErbB3/HER3, clone SP71 from Biomol [47] and the Dako envision Kit (Dako, Hamburg, Germany). The antibody was diluted 1:100 in Dako antibody diluent. Validation was done using duodenum as a positive control; isotype-specific immunoglobulin was used as a negative control (Supplementary Figure S5). Counterstaining was done using Papanicolaou's solution 1A Harris (Merck Millipore; Darmstadt; Germany). Slides were digitalized (SCN400 slide scanner; 20x objective; Leica Microsystems, Mannheim, Germany) and analyzed by a custom-made solution in the Tissue Studio software (Definiens, München, Germany), which was validated manually by two independent observers [48]. For each of the three cores of one patient specimen the percentage of cancer cells showing no, weak, moderate, and strong staining intensity was determined and averaged. Cores with less than 100 cancer cells were excluded from the analysis. For correlation analysis of ErbB3 expression and tumor grade, the cut-off for ErbB3 positivity was set between low and medium staining intensity. Tumor grades were determined as described [49]. For statistical analysis chi square trend test was performed using R (R Foundation for Statistical Computing, Vienna, Austria) with additional packages coin-1.0-24 [50].

### Statistical analysis

Data are expressed as mean ± S.E.M.; ‘n’ refers to the number of samples/experiment, N' to the number of independent experiments. Statistical significance was evaluated by t-test and one-way ANOVA followed by Tukey's post-test (GraphPad Prism version 4.03; GraphPad Software Inc., La Jolla, CA). p-values below 0.05 were considered significant (*p < 0.05; ** p < 0.01; *** p < 0.001; ns, p > 0.05).

## SUPPLEMENTARY MATERIALS FIGURES AND TABLE



## References

[1] Walther A, Johnstone E, Swanton C, Midgley R, Tomlinson I, Kerr D (2009). Genetic prognostic and predictive markers in colorectal cancer. Nat Rev Cancer.

[2] Brand TM, Wheeler DL (2012). KRAS mutant colorectal tumors: Past and present. Small GTPases.

[3] Barbacid M (1987). ras genes. Annu Rev Biochem.

[4] Brand TM, Iida M, Wheeler DL (2011). Molecular mechanisms of resistance to the EGFR monoclonal antibody cetuximab. Cancer Biol Ther.

[5] Normanno N, Tejpar S, Morgillo F, Luca A de, van Cutsem E, Ciardiello F (2009). Implications for KRAS status and EGFR-targeted therapies in metastatic CRC. Nat Rev Clin Oncol.

[6] Yarden Y, Sliwkowski MX (2001). Untangling the ErbB signalling network. Nat Rev Mol Cell Biol.

[7] Garrett TPJ, McKern NM, Lou M, Elleman TC, Adams TE, Lovrecz GO, Kofler M, Jorissen RN, Nice EC, Burgess AW, Ward CW (2003). The crystal structure of a truncated ErbB2 ectodomain reveals an active conformation, poised to interact with other ErbB receptors. Mol Cell.

[8] Amin DN, Campbell MR, Moasser MM (2010). The role of HER3, the unpretentious member of the HER family, in cancer biology and cancer therapeutics. Semin Cell Dev Biol.

[9] Guy PM, Platko JV, Cantley LC, Cerione RA, Carraway KL (1994). Insect cell-expressed p180erbB3 possesses an impaired tyrosine kinase activity. Proc Natl Acad Sci U S A.

[10] Baselga J, Swain SM (2009). Novel anticancer targets: revisiting ERBB2 and discovering ERBB3. Nat Rev Cancer.

[11] Breuleux M (2007). Role of heregulin in human cancer. Cell Mol Life Sci.

[12] Montero JC, Rodríguez-Barrueco R, Ocaña A, Díaz-Rodríguez E, Esparís-Ogando A, Pandiella A (2008). Neuregulins and cancer. Clin Cancer Res.

[13] Vermeer PD, Einwalter LA, Moninger TO, Rokhlina T, Kern JA, Zabner J, Welsh MJ (2003). Segregation of receptor and ligand regulates activation of epithelial growth factor receptor. Nature.

[14] Valastyan S, Weinberg RA (2011). Tumor metastasis: molecular insights and evolving paradigms. Cell.

[15] Gangarosa LM, Sizemore N, Graves-Deal R, Oldham SM, Der CJ, Coffey RJ (1997). A raf-independent epidermal growth factor receptor autocrine loop is necessary for Ras transformation of rat intestinal epithelial cells. J Biol Chem.

[16] Toulany M, Baumann M, Rodemann HP (2007). Stimulated PI3K-AKT signaling mediated through ligand or radiation-induced EGFR depends indirectly, but not directly, on constitutive K-Ras activity. Mol Cancer Res.

[17] Debnath J, Brugge JS (2005). Modelling glandular epithelial cancers in three-dimensional cultures. Nat Rev Cancer.

[18] Lee GY, Kenny PA, Lee EH, Bissell MJ (2007). Three-dimensional culture models of normal and malignant breast epithelial cells. Nat Methods.

[19] Sato T, Clevers H (2013). Growing self-organizing mini-guts from a single intestinal stem cell: mechanism and applications. Science.

[20] Jaffe AB, Kaji N, Durgan J, Hall A (2008). Cdc42 controls spindle orientation to position the apical surface during epithelial morphogenesis. The Journal of Cell Biology.

[21] Magudia K, Lahoz A, Hall A (2012). K-Ras and B-Raf oncogenes inhibit colon epithelial polarity establishment through up-regulation of c-myc. The Journal of Cell Biology.

[22] Möller Y, Siegemund M, Beyes S, Herr R, Lecis D, Delia D, Kontermann R, Brummer T, Pfizenmaier K, Olayioye MA (2014). EGFR-targeted TRAIL and a Smac mimetic synergize to overcome apoptosis resistance in KRAS mutant colorectal cancer cells. PLoS ONE.

[23] Armaghany T, Wilson JD, Chu Q, Mills G (2012). Genetic alterations in colorectal cancer. Gastrointest Cancer Res.

[24] Holbro T, Beerli RR, Maurer F, Koziczak M, Barbas CF, Hynes NE (2003). The ErbB2/ErbB3 heterodimer functions as an oncogenic unit: ErbB2 requires ErbB3 to drive breast tumor cell proliferation. Proc Natl Acad Sci U S A.

[25] Lee-Hoeflich ST, Crocker L, Yao E, Pham T, Munroe X, Hoeflich KP, Sliwkowski MX, Stern HM (2008). A central role for HER3 in HER2-amplified breast cancer: implications for targeted therapy. Cancer Research.

[26] Vaught DB, Stanford JC, Young C, Hicks DJ, Wheeler F, Rinehart C, Sánchez V, Koland J, Muller WJ, Arteaga CL, Cook RS (2012). HER3 is required for HER2-induced preneoplastic changes to the breast epithelium and tumor formation. Cancer Research.

[27] Jaiswal BS, Kljavin NM, Stawiski EW, Chan E, Parikh C, Durinck S, Chaudhuri S, Pujara K, Guillory J, Edgar KA, Janakiraman V, Scholz R, Bowman KK (2013). Oncogenic ERBB3 mutations in human cancers. Cancer Cell.

[28] Engelman JA, Zejnullahu K, Mitsudomi T, Song Y, Hyland C, Park JO, Lindeman N, Gale C, Zhao X, Christensen J, Kosaka T, Holmes AJ, Rogers AM (2007). MET amplification leads to gefitinib resistance in lung cancer by activating ERBB3 signaling. Science.

[29] Lee D, Yu M, Lee E, Kim H, Yang Y, Kim K, Pannicia C, Kurie JM, Threadgill DW (2009). Tumor-specific apoptosis caused by deletion of the ERBB3 pseudo-kinase in mouse intestinal epithelium. J Clin Invest.

[30] Steinkamp MP, Low-Nam ST, Yang S, Lidke KA, Lidke DS, Wilson BS (2014). erbB3 is an active tyrosine kinase capable of homo- and heterointeractions. Mol Cell Biol.

[31] van Schaeybroeck S, Kyula JN, Fenton A, Fenning CS, Sasazuki T, Shirasawa S, Longley DB, Johnston PG, van Schaeybroeck S, Kyula JN, Fenton A, Fenning CS, Sasazuki T (2011). Oncogenic Kras Promotes Chemotherapy-Induced Growth Factor Shedding via ADAM17 // Oncogenic Kras promotes chemotherapy-induced growth factor shedding via ADAM17. Cancer Research.

[32] Navas C, Hernández-Porras I, Schuhmacher AJ, Sibilia M, Guerra C, Barbacid M (2012). EGF receptor signaling is essential for k-ras oncogene-driven pancreatic ductal adenocarcinoma. Cancer Cell.

[33] Linch M, Sanz-Garcia M, Rosse C, Riou P, Peel N, Madsen CD, Sahai E, Downward J, Khwaja A, Dillon C, Roffey J, Cameron AJM, Parker PJ (2014). Regulation of polarized morphogenesis by protein kinase C iota in oncogenic epithelial spheroids. Carcinogenesis.

[34] Aranda V, Haire T, Nolan ME, Calarco JP, Rosenberg AZ, Fawcett JP, Pawson T, Muthuswamy SK (2006). Par6-aPKC uncouples ErbB2 induced disruption of polarized epithelial organization from proliferation control. Nat Cell Biol.

[35] Beji A, Horst D, Engel J, Kirchner T, Ullrich A (2012). Toward the prognostic significance and therapeutic potential of HER3 receptor tyrosine kinase in human colon cancer. Clin Cancer Res.

[36] Gespach C (2012). Increasing potential of HER3 signaling in colon cancer progression and therapy. Clin Cancer Res.

[37] Gala K, Chandarlapaty S (2014). Molecular pathways: HER3 targeted therapy. Clin Cancer Res.

[38] Yonesaka K, Zejnullahu K, Okamoto I, Satoh T, Cappuzzo F, Souglakos J, Ercan D, Rogers A, Roncalli M, Takeda M, Fujisaka Y, Philips J, Shimizu T (2011). Activation of ERBB2 signaling causes resistance to the EGFR-directed therapeutic antibody cetuximab. Sci Transl Med.

[39] Garrett JT, Olivares MG, Rinehart C, Granja-Ingram ND, Sánchez V, Chakrabarty A, Dave B, Cook RS, Pao W, McKinely E, Manning HC, Chang J, Arteaga CL (2011). Transcriptional and posttranslational up-regulation of HER3 (ErbB3) compensates for inhibition of the HER2 tyrosine kinase. Proc Natl Acad Sci U S A.

[40] Sergina NV, Rausch M, Wang D, Blair J, Hann B, Shokat KM, Moasser MM (2007). Escape from HER-family tyrosine kinase inhibitor therapy by the kinase-inactive HER3. Nature.

[41] Wheeler DL, Huang S, Kruser TJ, Nechrebecki MM, Armstrong EA, Benavente S, Gondi V, Hsu K, Harari PM (2008). Mechanisms of acquired resistance to cetuximab: role of HER (ErbB) family members. Oncogene.

[42] Sun C, Hobor S, Bertotti A, Zecchin D, Huang S, Galimi F, Cottino F, Prahallad A, Grernrum W, Tzani A, Schlicker A, Wessels LFA, Smit EF (2014). Intrinsic resistance to MEK inhibition in KRAS mutant lung and colon cancer through transcriptional induction of ERBB3. Cell Rep.

[43] Turke AB, Song Y, Costa C, Cook R, Arteaga CL, Asara JM, Engelman JA (2012). MEK inhibition leads to PI3K/AKT activation by relieving a negative feedback on ERBB receptors. Cancer Research.

[44] Röring M, Herr R, Fiala GJ, Heilmann K, Braun S, Eisenhardt AE, Halbach S, Capper D, Deimling A von, Schamel WW, Saunders DN, Brummer T (2012). Distinct requirement for an intact dimer interface in wild-type, V600E and kinase-dead B-Raf signalling. EMBO J.

[45] Sato T, Stange DE, Ferrante M, Vries RGJ, van Es JH, van den Brink S, van Houdt WJ, Pronk A, van Gorp J, Siersema PD, Clevers H (2011). Long-term expansion of epithelial organoids from human colon, adenoma, adenocarcinoma, and Barrett's epithelium. Gastroenterology.

[46] Ootani A, Li X, Sangiorgi E, Ho QT, Ueno H, Toda S, Sugihara H, Fujimoto K, Weissman IL, Capecchi MR, Kuo CJ (2009). Sustained *in vitro* intestinal epithelial culture within a Wnt-dependent stem cell niche. Nat Med.

[47] Wang S, Huang J, Lyu H, Cai B, Yang X, Li F, Tan J, Edgerton SM, Thor AD, Lee CK, Liu B (2013). Therapeutic targeting of erbB3 with MM-121/SAR256212 enhances antitumor activity of paclitaxel against erbB2-overexpressing breast cancer. Breast Cancer Res.

[48] Fisel P, Stühler V, Bedke J, Winter S, Rausch S, Hennenlotter J, Nies AT, Stenzl A, Scharpf M, Fend F, Kruck S, Schwab M, Schaeffeler E (2015). MCT4 surpasses the prognostic relevance of the ancillary protein CD147 in clear cell renal cell carcinoma. Oncotarget.

[49] Fux R, Schwab M, Thon K, Gleiter CH, Fritz P (2005). Cyclooxygenase-2 expression in human colorectal cancer is unrelated to overall patient survival. Clin Cancer Res.

[50] Hothorn T, Hornik K, van de Wiel, Mark A, Zeileis A (2006). A Lego System for Conditional Inference.

