# Supplementary material for: Oncogenic Ras triggers hyperproliferation and impairs polarized colonic morphogenesis by autocrine ErbB3 signaling
Source: Oncotarget. 2016 Jul 18;7(33):53526–39. doi: 10.18632/oncotarget.10658 (PMC5288203; doi:10.18632/oncotarget.10658)
Supplement: Supplementary file 1 [file oncotarget-07-53526-s001.pdf]

Oncogenic Ras triggers hyperproliferation and impairs polarized colonic morphogenesis by autocrine ErbB3 signaling

SUPPLEMENTARY DATA

REFERENCE

1. Grimm C, Chavez L, Vilardell M, Farrall AL, Tierling S, Böhm JW, Grote P, Lienhard M, Dietrich J, Timmermann B, Walter J, Schweiger MR, Lehrach H, et al. DNA-methylome analysis of mouse intestinal adenoma identifies a tumour-specific signature that is partly conserved in human colon cancer. PLoS genetics 2013; 9:e1003250.

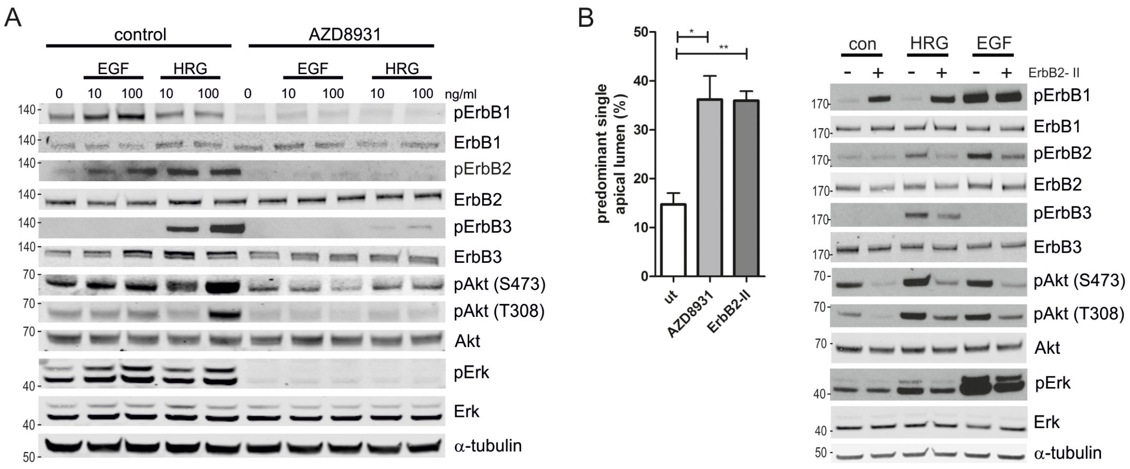

Supplementary Figure S1: ErbB receptor inhibition by AZD8931 and ErbB2-II. A. Caco-2 cells were seeded in 2D, serum-starved overnight (2% FCS) and, treated with AZD8931 (200 nM) for 1 h prior to stimulation with the indicated growth factors (10 ng/ml or 100 ng/ml) for 15 min or left untreated (control). Cells were lysed and analyzed by immunoblotting using the indicated antibodies (pErbB2 (Tyr1221/1222)). Tubulin was detected as a loading control. Shown is a representative blot (N=2). (B, left panel) Caco-2tet K-Ras^{G12V} cells were seeded into 3D cultures. One day post seeding K-Ras^{G12V}-expression was induced by dox and cultures were left untreated or treated with 100 μM ErbB2-II. Two days later CTX was added. Cultures were fixed the next day and stained with DAPI (nuclei; blue) and phalloidin (F-actin; red) (data not shown). The percentage of cysts with PSAL from was determined (n>60; N=3). (B, right panel) Caco-2 cells were seeded in 2D, serum-starved overnight (2% FCS) and, treated with ErbB2-II (100 μM) for 1 h prior to stimulation with the indicated growth factors (10 ng/ml) for 15 min or left untreated (control). Cells were lysed and analyzed by immunoblotting using the indicated antibodies. Tubulin was detected as a loading control.

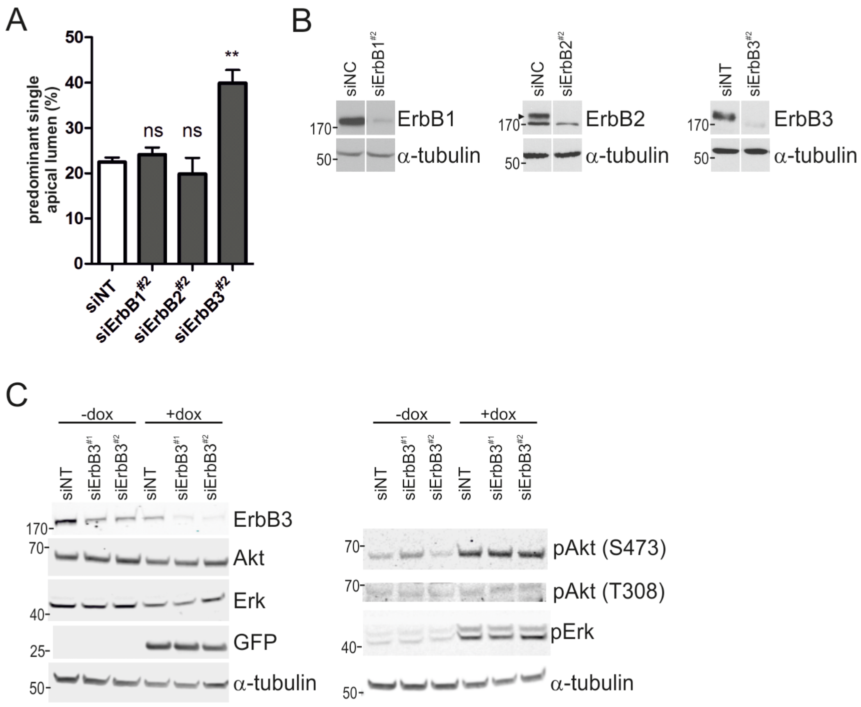

Supplementary Figure S2: ErbB3 knockdown rescues polarized morphogenesis of Caco-2 cells expressing oncogenic K-Ras. A. Caco-2tet K-Ras^{G12V} cells were transfected with non-targeting (siNT) and ErbB-receptor specific siRNAs (#2), respectively. The next day cells were seeded into 3D culture. One day post seeding K-Ras^{G12V} expression was induced with dox. Two days later lumen expansion was induced by addition of 100 ng/ml CTX. The next day, cultures were fixed and stained with DAPI and phalloidin (not shown). The percentage of cysts with PSAL was determined (n>70; N=3). **B.** 48 h after gene silencing, lysates were generated and analyzed by immunoblotting using the indicated antibodies. Tubulin was detected as a loading control. Specific bands are marked by arrowheads. In all cases, corresponding control and knockdown lysates were analyzed on the same blot. **C.** Caco-2tet K-Ras^{G12V} cells were transfected with non-targeting (siNT) and two independent ErbB3-specific siRNAs (#1, #2), respectively. The next day, cells were seeded into 3D culture and K-Ras^{G12V} expression was induced with dox one day later. Three days later, cysts were isolated and lysed. Lysates were analyzed by immunoblotting using the indicated antibodies. Tubulin was detected as a loading control. Shown is a representative blot (N=2).

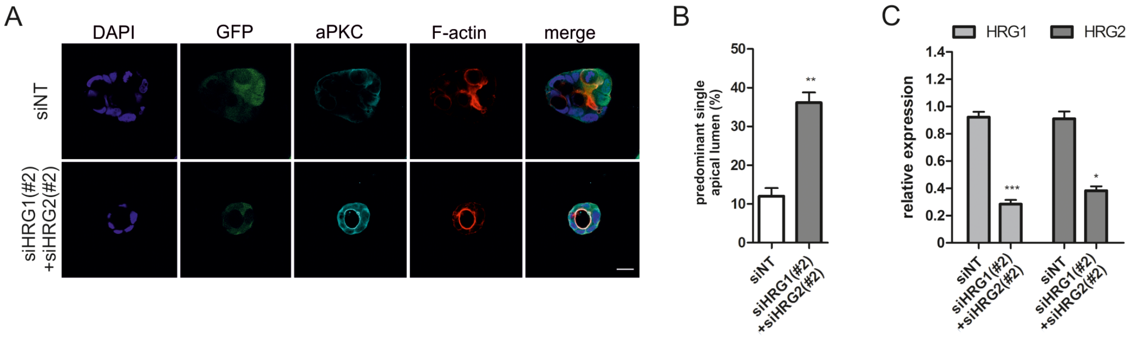

Supplementary Figure S3: HRG knockdown by independent siRNAs in K-Ras^{G12V} expressing Caco-2 cells. (A+B) Caco-2tet K-Ras^{G12V} cells were transfected with non-targeting (siNT) and a mix of HRG1- and HRG2-specific siRNAs (#2). The next day cells were seeded into 3D culture and K-Ras^{G12V} expression was induced with dox one day later. Two days after seeding CTX was added. Cultures were fixed the next day and stained with DAPI (nuclei; blue), anti-aPKC antibody (cyan) and phalloidin (F-actin; red). GFP is coexpressed with K-Ras^{G12V} (green). **A.** Shown are confocal sections of the midplane of representative cysts (scale: 20 µm). **B.** The percentage of cysts with PSAL from (B) was determined (n>70; N=3). **C.** Two days after gene silencing, RNA of the transfected cells was extracted. *HRG* levels were determined by qPCR and normalized to *GAPDH* (N=3).

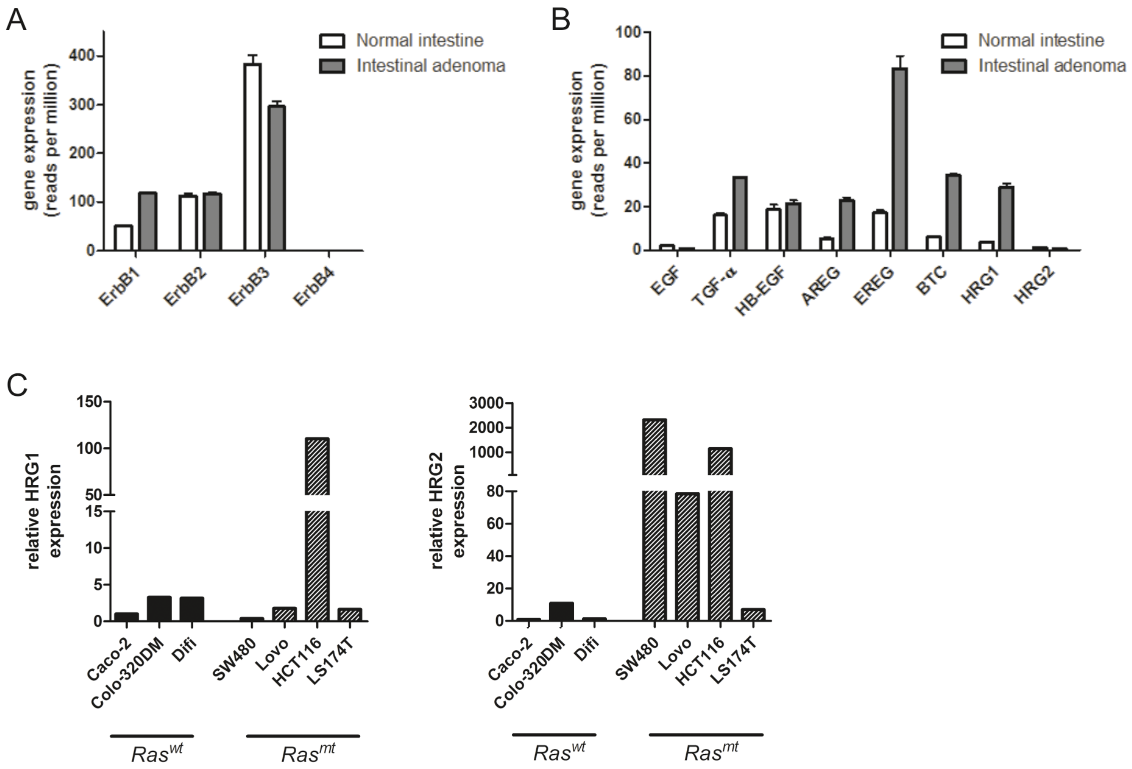

Supplementary Figure S4: Expression of genes encoding ErbB receptors and their ligands in the normal intestinal epithelium and in intestinal adenoma of the mouse and in human CRC cell lines. A. *EGFR*, *ErbB2* and *ErbB3*, but not *ErbB4*, are expressed in the normal intestine and in adenoma. **B.** Several ErbB ligands including *HRG1* are upregulated in adenoma. (A+B) Graphs show mean values of at least three biological replicates. RNAseq data are available as part of GEO data set GSE38983 (1). (C) The indicated human CRC cell lines were seeded into 3D culture. Three days later, cysts were isolated, RNA was extracted and *HRG1/HRG2* expression was determined by qPCR and normalized to *GAPDH* (N=2).

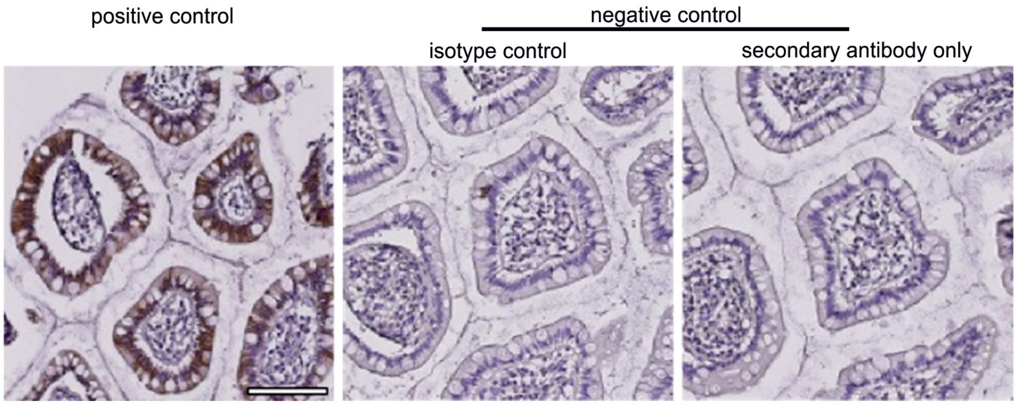

Supplementary Figure S5: Immunohistochemical validation of ErbB3 clone SP71. ErbB3 staining of duodenal tissue serving as a positive control. Negative controls were performed by using the isotype-specific immunoglobulin (middle) and by omitting the primary antibody (right). (scale: 100 µm)

Supplementary Table S1: Patient and clinicopathological data of the analyzed tumor samples

	mean (+/- sd)
Age	68.76 (+/- 10.95)
Sex	
male	197 (49.4%)
female	202 (50.6%)
Stage	
T1	15 (3.8%)
T2	88 (22.1%)
T3	267 (66.9%)
T4	26 (6.5%)
not available	3 (0.8%)
Nodal	
N0	225 (56.4%)
N1	121 (30.3%)
N2	51 (12.8%)
not available	2 (0.5%)
Metastasis	
M0	373 (93.5%)
M1	23 (5.8%)
not available	3 (0.8%)
Grade	
G1	27 (6.8%)
G2	299 (74.9%)
G3	73 (18.3%)
Localization	
Coecum	50 (12.5%)
Colon ascendens	53 (13.2%)
Flexur dextra	22 (5.5%)
Colon transversum	37 (9.3%)
Flexur sinistra	18 (4.5%)
Colon descendens	31 (7.8%)
Sigma	185 (46.4%)
multifocal	3 (0.8%)
